# Long-Term Creep Rupture of Carbon Fiber Reinforced Polymer Grids Under High Stress Levels: Experimental Investigation

**DOI:** 10.3390/ma18010035

**Published:** 2024-12-25

**Authors:** Menghay Phoeuk, Dong-Yeong Choi, Minho Kwon

**Affiliations:** Department of Civil Engineering, Gyeongsang National University, Jinju 52828, Republic of Korea; menghayph@gnu.ac.kr (M.P.); ced5189@gnu.ac.kr (D.-Y.C.)

**Keywords:** CFRP grids, creep rupture strength, creep reduction factor, durability, long-term performance

## Abstract

Corrosion in reinforced concrete (RC) structures has led to the increased adoption of non-corrosive materials, such as carbon fiber-reinforced polymers (CFRPs), as replacements for traditional steel rebar. However, ensuring the long-term reliability of CFRP grids under sustained stress is critical for achieving safe and effective designs. This study investigates the long-term tensile creep rupture behavior of CFRP grids to establish a design threshold for their tensile strength under sustained loading conditions in demanding structural applications. A comprehensive laboratory experiment was conducted over 10,000 h, during which CFRP grid specimens were subjected to constant stress levels ranging from 92% to 98% of their ultimate tensile strength. The results confirm the excellent creep rupture resistance of CFRP grids. Specimens subjected to a sustained stress ratio of 92% of their ultimate tensile strength remained intact throughout the testing period, with minimal creep strain ranging from approximately 1% to 4% of the initial strain. The mean extrapolated creep rupture factors were found to be 92.1% and 91.7% of their ultimate tensile strength for service lives of 50 and 114 years, respectively. Based on the results of this study, a tensile stress limit of 48% of the ultimate tensile strength is recommended for CFRP grids to ensure long-term creep rupture resistance over a 100-year service life.

## 1. Introduction

The ongoing investigation forms a segment of a broader series aimed at enabling the utilization of locally manufactured carbon fiber-reinforced polymer (CFRP) grids as internal reinforcement within reinforced concrete (RC) members. As part of this endeavor, the fundamental properties essential for establishing design thresholds for tensile stress resisting sustained load are clarified in this study.

Fiber-reinforced polymer (FRP) materials consist of fibers embedded in a resin matrix, with their mechanical properties—such as strength and elastic modulus—determined by the type and proportions of their constituent materials. These composites offer significant advantages over traditional steel, including higher strength, lighter weight, and superior corrosion resistance [1,2,3]. However, their brittle nature necessitates careful consideration in structural applications to mitigate potential vulnerabilities [1,2].

Over the past two decades, FRP composite bars have gained widespread acceptance in construction due to their high specific strength, lightweight properties, and resistance to corrosion [3]. FRP bars are categorized into glass fiber-reinforced polymer (GFRP), Basalt Fiber Reinforced Polymer (BFRP), carbon fiber-reinforced polymer (CFRP), and aramid fiber-reinforced polymer (AFRP), depending on the type of fiber. Table 1 compares the fundamental mechanical tensile properties of steel and FRP reinforcing bars. Design and construction recommendations for structures reinforced with these materials are provided in standards such as the American Concrete Institute (ACI) Committee Report 440.1R [1] and various regional guidelines, which emphasize their demanding application in construction.

Among the different types of FRP, CFRP stands out for its exceptional performance characteristics, particularly in applications requiring superior strength-to-weight ratios, stiffness and durability [4,5]. The increasing adoption of CFRP as a replacement for steel reinforcement in RC structures is driven by its unparalleled corrosion resistance and mechanical superiority [5]. Compared to GFRP, BFRP and AFRP, CFRP exhibits higher tensile strength, lower density, and better long-term performance under environmental and mechanical stressors [1,5,6].

Despite its many advantages, the successful application of CFRP requires a comprehensive understanding of its time-dependent behavior, particularly creep rupture—gradual failure under sustained loading—which can compromise structural integrity [3,6]. These challenges are especially critical in high-durability applications, where prolonged loading can lead to significant performance degradation over time. Therefore, a thorough investigation of CFRP’s long-term behavior under varying stress conditions is vital to ensuring its safe and reliable application in RC structures.

### 1.1. ACI 440.1R Design Consideration

The behavior of FRP composite materials remains linearly elastic up to failure, offering significantly higher tensile strength than steel rebars but with lower stiffness, as summarized in Table 1. Due to that, FRP-reinforced RC members exhibit distinct performance characteristics compared to those with conventional steel reinforcement. Given that FRP materials tend to fail suddenly without prior yielding, design approaches for FRP-reinforced RC members are generally based on strength and working stress principles.

To control the working stress in FRP, ACI 440.1R [1] introduces two reduction factors: the environmental reduction factor ($C_{E}$), which accounts for exposure conditions, and the creep rupture reduction factor ($C_{C}$), which addresses material failure under sustained stress. These factors are applied together to the guaranteed tensile strength ($f_{fu}^{*}$) of FRP materials, defined as the average ultimate tensile strength ($f_{u,ave}$) minus three standard deviations ($f_{fu}^{*}=f_{u,ave}-3\sigma$). The design tensile strength ($f_{fu}$) is then calculated by applying the environmental reduction factor ($C_{E}$) to the guaranteed tensile strength ($f_{fu}^{*}$), as shown in Equation (1):

(1) $$f_{fu}=C_{E}\times f_{fu}^{*}$$

Similarly, the design rupture strain ($\varepsilon_{fu}$) is calculated as follows:

(2) $$\varepsilon_{fu}=C_{E}\times \varepsilon_{fu}^{*}$$

The design modulus of elasticity is assumed to be the same as the value reported by the manufacturer, which is the mean elastic modulus (guaranteed value) of a sample of test specimens ($E_{f}=E_{f,ave}$).

In this context, the environmental reduction factor ($C_{E}$) accounts for the impact of environmental exposure on the tensile strength of FRP materials over the structure’s service life. The value of $C_{E}$ can be determined either from recommended design provisions or from performance-based durability tests conducted in specific simulated environments.

The unconditioned creep rupture strength ($f_{fc}$) of FRP materials is obtained by applying the creep reduction factor to $f_{fu}^{*}$, as shown in Equation (3):

(3) $$f_{fc}=C_{C}\times f_{fu}^{*}$$

The creep reduction factor $C_{C}$ can be determined by conducting long-term laboratory creep rupture tests.

For design purposes, the stress limit of FRP materials under sustained service loads in exposed conditions ($f_{fs,sus}$) is calculated by applying both the environmental reduction factor ($C_{E}$) and the unconditioned creep rupture reduction factor ($C_{C}$) to $f_{fu}^{*}$, as shown in Equation (4):

(4) $$f_{fc}=C_{E}\times C_{C}\times f_{fu}^{*}$$

To meet service design criteria, the sustained stress requirement is satisfied by ensuring that the calculated tensile stress in FRP materials ($f_{t,sus}$), derived from unfactored sustained load, remains within the limit $f_{fs,sus}$, as expressed in Equation (5):

(5) $$f_{t,sus}\leq f_{fs,sus}$$

To ensure durability and prevent premature failure when using FRP materials as internal reinforcement in RC members, it is essential to evaluate the factors that reduce their tensile strength, such as creep rupture and exposure to harsh environmental conditions [1,6]. This evaluation is critical for minimizing the risk of early structural failure and enhancing engineers’ confidence in fully leveraging the capabilities of FRP materials. By addressing these factors, the safety of the structure can be assured, and the economic benefits of FRP reinforcement can be maximized.

### 1.2. Existing Long-Term Creep Ruptures Test Methods of FRP Materials

Creep rupture in FRP materials refers to their tensile failure under prolonged, constant loading. When designing RC members with FRP reinforcement, the creep rupture strength of the FRP is a critical parameter for defining the allowable sustained stress during the service life [1]. Various international testing guidelines have been developed to assess creep rupture, as listed in Table 2. These testing methods typically follow similar procedures to determine the creep rupture strength factor of FRP materials.

To evaluate the creep rupture properties, FRP specimens are subjected to varying load levels, expressed as percentages of their ultimate tensile strength, while recording the corresponding time-to-rupture for each prescribed load level [7,8,9,10]. The data points obtained are plotted on a semi-logarithmic graph, with the stress level, $Y_{c}\;\left(\%\right)$, on the vertical axis and the logarithm of time, log(t), on the horizontal axis. Using the least squares method, a best-fit linear curve is derived, represented by Equation (6):

(6) $$Y_{c}=a_{1}-b_{1}\times \log\left(t\right)$$

Fitting Equation (6) to experimental data establishes a logarithmic relationship between the load ratio and time-to-rupture, a commonly used model for characterizing creep behavior [9]. The creep rupture strength factor for a specific time frame can be determined by extrapolating this curve. For instance, to find the creep rupture factor corresponding to a one-million-hour, *t* = 1,000,000 h is substituted into the obtained equation. This value represents the one-million-hour creep rupture factor for the investigated FRP material.

The corresponding load, known as the one-million-hour creep rupture capacity, is calculated using the following formula:

(7) $$F_{r}=Y_{c}\left(t=\mathrm{one}\;\mathrm{million}\;\mathrm{hours}\right)\times F_{fu}$$

where $F_{fu}$ is the ultimate tensile load of the FRP, and $F_{r}$ is the one-million-hour creep capacity. The one-million-hour creep strength, $f_{r}$, is then determined by dividing $F_{r}$ by the cross-sectional area, $A_{f}$, of FRP material.

### 1.3. Previous Studies on CFRP Creep

Unlike steel reinforcing bars, CFRP materials are susceptible to creep rupture under sustained stress, even at levels below their static tensile strength [9]. To mitigate this risk, design guidelines set specific stress limits to prevent long-term failure [1,11,12,13,14]. For example, ACI 440.1R [1] recommends a design creep rupture stress limit of 55% of the design tensile strength for CFRP bars, aimed at ensuring structural integrity for up to 50 years. However, this criterion, based on limited experimental data for endurance times beyond 100 h, introduces uncertainty into the design process. While conservative stress limits may enhance safety, they also restrict the efficient use of CFRP materials under sustained loads. The creep rupture coefficient plays a crucial role in the design of structures exposed to prolonged stresses, such as retaining walls or heavy storage facilities. This highlights the need for long-term experimental studies to better understand CFRP creep behavior. Such data would not only refine the design coefficient, improving economic efficiency, it would also instill greater confidence among practitioners in the safe and optimal use of CFRP materials in engineering applications.

The creep behavior of CFRP materials is significantly influenced by the time temperature-dependent properties of both the carbon fibers and the resin matrix, a phenomenon commonly known as viscoelastic behavior [15,16,17,18]. The creep characteristics of CFRP materials can vary notably based on the type of carbon fiber, the epoxy resin used, the fiber content, as well as the time, temperature, and level of sustained load. To date, various authors [17,19,20,21] have used predictive models like Findley and Burger to characterize the viscoelastic behavior of CFRP composites over time. They adopted the results from laboratory creep tests on various types of CFRP materials into these models, which offer valuable frameworks for understanding CFRP performance under sustained loading conditions and provide insights into their creep behavior. For instance, Almeida Jr. [19] demonstrated that Findley’s model accurately predicts the creep behavior of carbon fiber-reinforced epoxy filament wound laminates at high temperatures. On the other hand, Majda et al. [20] found that the Burger model, which incorporates Maxwell and Kelvin–Voigt elements, provides a precise description of the creep behavior of epoxy adhesives at ambient temperatures, making it particularly suitable for environments with minimal temperature fluctuations. While these studies provide invaluable insights into CFRP creep behavior, they are limited by the short duration of investigation and low sustained stress levels. These constraints may not accurately reflect the long-term stress that CFRP experiences in practical scenarios, where the material is typically subjected to higher stress over extended periods matching the design life of structures. Therefore, it is important to investigate the creep behavior of CFRP under higher stress and longer testing duration to establish allowable stress levels to ensure the material performs reliably under sustained loads over extended periods.

Recent studies on the long-term creep behavior of CFRP materials under high stress ratios have been conducted by performing long-term creep experiments on various types of CFRP materials, such as pultruded laminated plates [22] and tendons [23]. These investigations involved continuously subjecting specimens to sustained stress levels ranging from 15% to 85% of their respective ultimate static tensile strength. During the testing period, none of the specimens ruptured, indicating that CFRP composite materials perform exceptionally well under sustained loading, suggesting a high creep rupture threshold. In the case of CFRP pultruded plates, which were subjected to sustained stress at 75% of their ultimate tensile strength for over 76 days (approximately 1800 h), they exhibited a creep strain of only 1.93%, deviating from the initial strain. Similarly, for CFRP tendons, applying the highest load ratio of 85% of their ultimate tensile strength resulted in a creep of just 1.16% from the initial strain over a test period of 1000 h. While these investigations provide valuable background into the long-term creep characteristics of CFRP materials under high sustained stress levels, they are unable to establish the creep rupture design factor. To determine the creep rupture factor and establish the stress limit for design purposes, additional creep rupture tests—tests designed to determine the time to rupture under constant loads—are essential. Long-term creep rupture tests are necessary to ascertain the creep reduction factor for establishing a safe design margin without compromising structural integrity when using CFRP materials as internal reinforcement in RC structures [9,10], which will help achieve both confidence for practitioners and an economically viable design.

Recently, Grace et al. [24] conducted an extensive long-term creep rupture investigation on CFRP materials. The study involved CFRP strands with diameters of 0.6 inches and 0.7 inches (15 mm and 18 mm), which were subjected to a testing period of 1000 h. During the test period, the specimens were continuously loaded up to 90% of their ultimate tensile strength. Some of the tested specimens reached rupture, while others, which did not rupture after 1000 h, were included in the analysis. The data from both ruptured and non-ruptured specimens were combined to establish a regression line, following the methodology outlined in Section 1.2. From this extrapolation, the minimum one-million-hour creep rupture strength of CFRP strands was estimated to be 88% of their tensile strength. The one-million-hour creep rupture factor derived from this experiment is considered conservative, as it includes non-ruptured specimens in the extrapolation. This results in a lower creep rupture factor, leading to a more conservative design approach.

### 1.4. Research Significance

This study focuses on experimentally assessing the long-term creep rupture behavior of CFRP grids under high sustained stress levels, approaching their static tensile strength. The primary objective is to establish a design threshold factor, referred to as the creep rupture stress limit factor, for their use as internal reinforcement in RC structures. Unlike prior research, which often overlooks the long-term creep performance of CFRP grids under high sustained stress, this work introduces an extensive tensile creep rupture test program spanning over 10,000 h, more than one year. Throughout this period, CFRP grid specimens were subjected to constant high-stress levels, up to 98% of their tensile strength, in controlled laboratory conditions. The resulting findings will not only offer knowledge about the behavior of CFRP grids under sustained loading but will also contribute to expanding the creep rupture literature database for longer endurance times. Furthermore, these results will enable the determination of a one-million-hour creep rupture factor. Based on these findings, a reduction factor will be proposed to establish a reliable design threshold for tensile strength under long-term loading conditions.

## 2. Materials and Methods

### 2.1. Materials and Specimen Preparation

**Figure 1:**
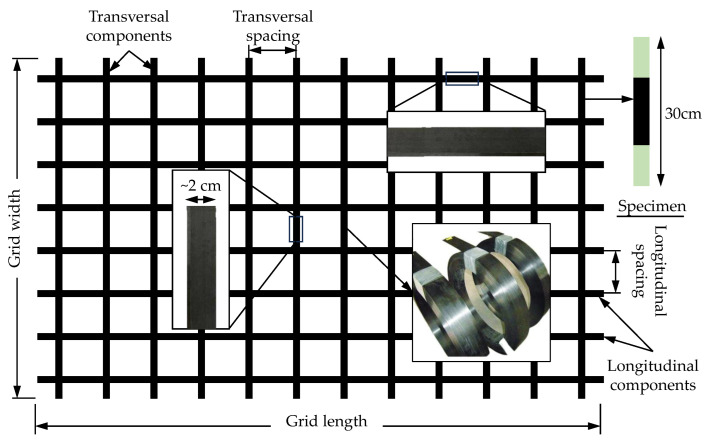
An overview of the description of CFRP grids.

The CFRP grids employed in this research were supplied by KCarbon (Republic of Korea). Figure 1 provides an overview of the CFRP grids being evaluated. These CFRP grids come in diverse width and length configurations, along with adjustable grid spacing. The grid’s components, approximately 20 mm in width and 1 mm in thickness with a smooth surface were fabricated using pultrusion technology, incorporating carbon fibers and epoxy resin. Table 3 outlines the key properties of the CFRP grids utilized in the creep testing program. The values listed for effective ultimate strain and ultimate load are derived from the tensile testing of five specimens performed following the procedure outlined in ASTM D3039 [25] standards.

**Figure 2:**
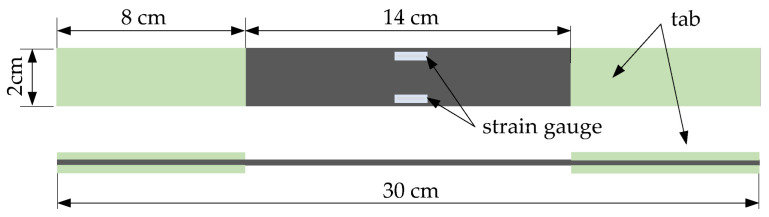
Detail dimension of CFRP grid specimen for tensile and creep test.

Conducting experiments on CFRP grids, as shown in Figure 1, is highly desirable for practical applications. However, performing creep rupture tests on full-size CFRP grids using large-scale testing equipment would be both time-consuming and costly. To address this challenge, this study adopted a more efficient approach by utilizing unidirectional CFRP grid specimens, as illustrated in Figure 2, which focus on a single direction of the CFRP grid components. Each creep specimen was prepared by cutting a 30 cm segment from a lengthier CFRP grid and attaching tabs to both ends using a high-bond adhesive material. The tab length was determined through estimation and preliminary testing to prevent slippage during testing. The specimens for both static tensile and creep tests were prepared in the same manner, with the dimensional details provided in Figure 2.

### 2.2. Long-Term Tensile Rupture Creep Test Setup

**Figure 3:**
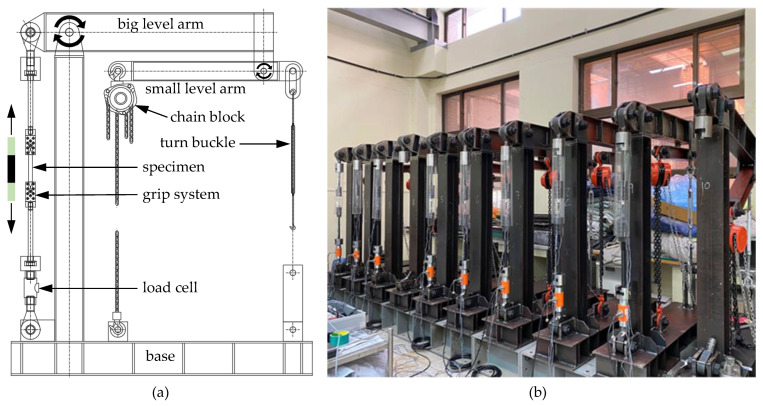
Creep test frame: (**a**) Schematic of the reaction frame and (**b**) Photograph of Creep rupture test program.

In this experiment, reaction frames equipped with a two-level arm adjustment system, as shown in Figure 3, were used to maintain nearly constant tensile stress on the specimens throughout the test period. Before fabrication, the reaction frame was simulated using the commercial FEM package Abaqus v18 to ensure all components stayed within the elastic range of material behavior. This helped minimize any small deformations and ensured the frame could maintain a constant sustained stress on the specimens. During the setup, each specimen was securely gripped at both ends and attached to the reaction frame, after which the load was applied.

To monitor creep strain, three electrical strain gauges were affixed longitudinally to each specimen, and aligned with the fiber direction. These gauges were connected to a data logger programmed to automatically record strain data at fixed time intervals. Additionally, an electronic load cell with a 500 kN capacity, connected to the same data logger was used to monitor the applied load throughout the test (as shown in Figure 3). This setup allowed for adjustment of the applied load if any rotation occurred in the frame lever arms.

During the testing, as creep strain developed in the CFRP grid specimens, the level arm of the test frame rotated, resulting in a decrease in the applied load. Manual adjustments—by rotating the turnbuckle—were made to ensure that the specimens remained subjected to a constant load within a tolerance of ±1% of the desired sustained force, as specified by ASTM D7337 [10].

Before loading, each specimen was carefully aligned vertically to minimize any eccentric loading. Once aligned, the load was gradually applied by incrementally rotating the turnbuckle until the target load was reached. Great attention was given during this process to avoid any sudden shocks to the specimen, which could lead to immediate failure. After loading, the applied load was closely monitored and maintained near the target value until the specimen ruptured. Simultaneously, strain data were continuously recorded using the time-step data logger.

For the creep test program, load ratios at four levels—98%, 96%, 94%, and 92% of the ultimate tensile strength—were selected, with equal intervals between them. Prior to establishing these load ratios, several preliminary tests were conducted to ensure that specimens at the highest load ratio ruptured after more than one hour, in line with ASTM D7337 recommendations [10]. This specification was intended to minimize the impact of the initial loading ramp on creep rupture time and ensure that all specimens experienced creep. The small interval between load ratios was chosen based on existing literature to ensure that some specimens subjected to the three highest load ratios would reach rupture. This would allow for the establishment of a regression model for predicting creep rupture strength. Using larger intervals could result in some specimens not reaching rupture at lower load ratios, leading to insufficient data for effective regression and creep rupture strength prediction.

## 3. Results and Discussion

### 3.1. Experimental Results on Creep Rupture of CFRP Grids

The creep test program in this study followed the procedure outlined in ASTM D7337 [10]. CFRP grid tensile creep rupture tests have been conducted for over 10,000 h under laboratory environment conditions. Upon completion of the tests, unbroken specimens were removed from the test frame, and a tensile test was performed to examine their retention properties. The creep test program along with the corresponding experimental outcomes sorted by the load ratios are summarized in Table 4.

The obtained results reveal significant creep resistance in the investigated CFRP grids. Throughout the testing period, for instance, all specimens subjected to sustained stress levels within 92% of their static tensile strength remained intact, indicating high creep rupture resistance. This observation is consistent with previous research on the long-term creep behavior of CFRP materials [22,23,24].

Moreover, the investigated CFRP grids exhibited minimal creep strain under sustained loading conditions. It was observed that when subjected to sustained loads greater than 94% of their ultimate strength, specimens experienced an accelerated creep rate, potentially leading to failure. However, for sustained loads below 94% of their ultimate strength, specimens developed very low creep strain and did not rupture during the test period. Among the tested specimens that reached rupture, creep strain ranged from 4 to 13% of the initial strain for sustained stress levels between 94% and 98% of their static tensile strength, as shown in Table 4 for specific details. Additionally, specimens subjected to load ratios between 92% and 94% of their ultimate load developed very low creep strain—approximately 1% to 4% of their respective initial strain—over a period exceeding 10,000 h, indicating minimal creep strain development.

To assess the impact of sustained stress on the CFRP grid’s mechanical properties, all unbroken specimens performed tensile tests. As detailed in Table 5, residual tensile strength ranged from 98% to 103% of their original strength, while the average after-creep elastic modulus experienced a 0.8% increase to 145.36 GPa, and the rupture strain decreased by 1% of their original rupture stain, decreasing from 19,770.30 με to 19,575.14 με. Additionally, no significant trend was found in the residual mechanical properties between specimens subjected to sustained load ratios of 92% and 94%, potentially due to the narrow load ratio intervals utilized in testing. However, with the average values obtained from the specimens subjected to sustained loads of 92% and 94% over a period of 10,000 h, the residual mechanical properties of CFRP grids remained nearly unchanged compared to their original properties. This suggests a resilient performance in resisting sustained loading of the investigated CFRP grids.

### 3.2. Observed Failure of the CFRP Tested Specimens

**Figure 4:**
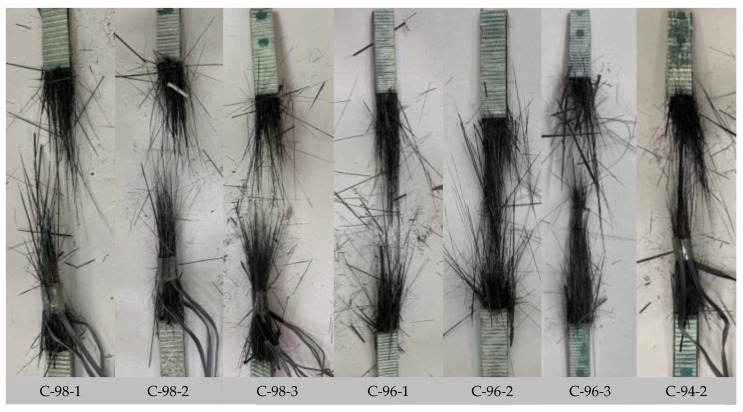
Failure mode of CFRP grid specimens during creep tests.

The failure modes of CFRP grid specimens under sustained stress were thoroughly examined. The specimens exhibited typical failure characteristics, with ruptures consistently occurring in the central region of the specimens, as shown in Figure 4. This behavior aligns with the expected failure patterns specified in ASTM D3039 [25], confirming proper specimen preparation and adherence to testing protocols. Undesirable failure modes, such as ruptures at the grip section, slippage, or other anomalies that could introduce bias, were not observed.

The CFRP grid specimens fractured abruptly with a dull sound after sustaining a constant load for the duration specified in Table 4. Despite variations in applied stress, the failure patterns remained uniform across all specimens. All fibers failed in a brittle manner within the middle gauge section, mirroring the results from static tensile tests. This consistency further validates that the failure modes were unaffected by setup issues or grip-related failures, ensuring the reliability of the long-term creep rupture results.

### 3.3. Creep Strain–Time Curve of CFRP Grids Under Sustained Loading

**Figure 5:**
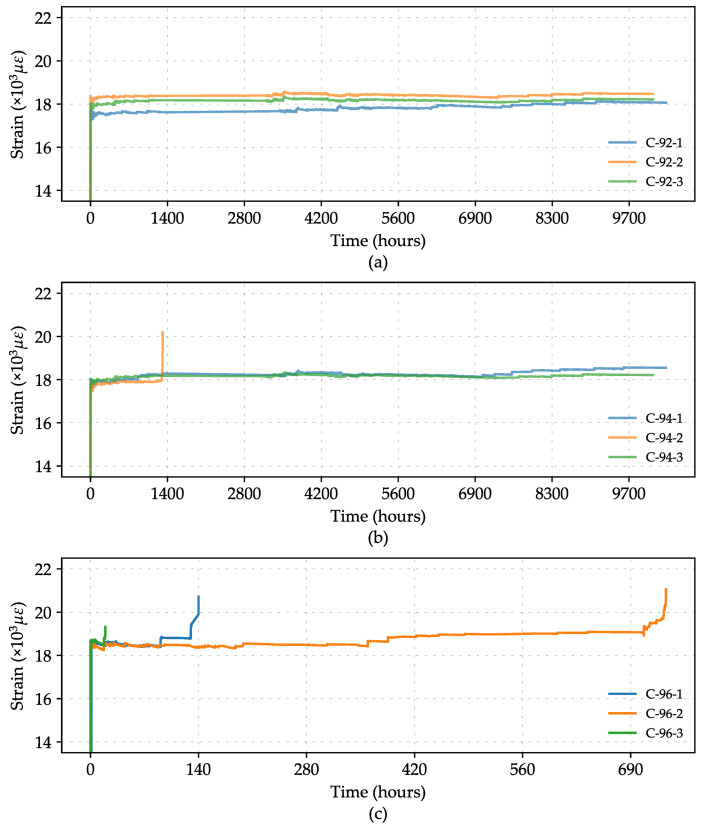

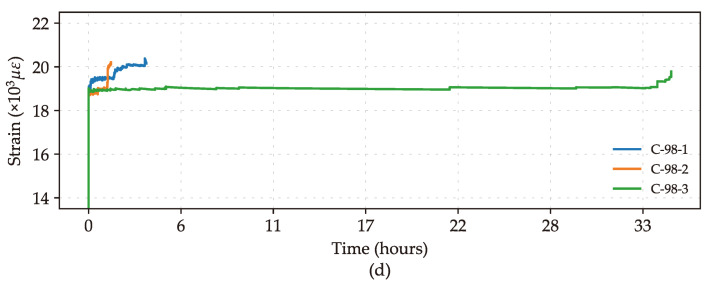
Strain vs. Time recorded during Creep test program for specimens subjected to (**a**) 92% Load Ratio, (**b**) 94% Load Ratio, (**c**) 96% Load Ratio, and (**d**) 98% Load Ratio.

Throughout the test period, the CFRP grids exhibited distinct behavior. They showed minimal secondary creep strain, indicating their capacity to withstand sustained loading with minimal deformation, as demonstrated in Figure 5. However, the CFRP grid specimens also showed an unexpected tendency for immediate failure when subjected to initial stress levels exceeding the creep-rupture threshold. Specifically, at a 98% load ratio, the CFRP grid specimens exhibited rupture times ranging from 1 h to 35 h. At a 96% load ratio, rupture times varied from 19 h to 739 h, while at 94%, rupture times spanned from 1300 h to over 10,000 h, with some specimens not rupturing at all, as shown in Table 4. On the other hand, no failures occurred at a 92% load ratio within the test duration. Notably, significant variation in rupture times was observed even at the same load ratio. This phenomenon is not unique to this study, similar large variations in rupture times for CFRP materials have been reported in previous studies [24], as well as for other FRP materials such as GFRP [26] and BFRP [27]. This variability has led to the adoption of a logarithmic time scale versus load ratios in various testing standards and guidelines, including those from JSCE, CSA, ACI, and ASTM, to more accurately predict creep rupture behavior.

These observations lead to an important conclusion: the threshold for creep rupture in CFRP grids occurs around 92% of their static tensile strength. This marks a critical limit for operational safety and structural design considerations for CFRP composite materials. Understanding the behavior of CFRP composites under sustained loading, especially when stress levels approach their static tensile strength, is essential for designing durable structures that can withstand long-term loading without compromising safety or performance. These results have significant implications, particularly in areas where maintaining structural integrity under sustained loading is crucial, such as heavy-duty storage facilities and industrial equipment support systems. By thoroughly understanding the complexities of creep rupture in CFRP composites, engineers can develop strategies to improve structural reliability and durability in critical applications.

### 3.4. One-Million Creep Rupture Strength Calculation

**Figure 6:**
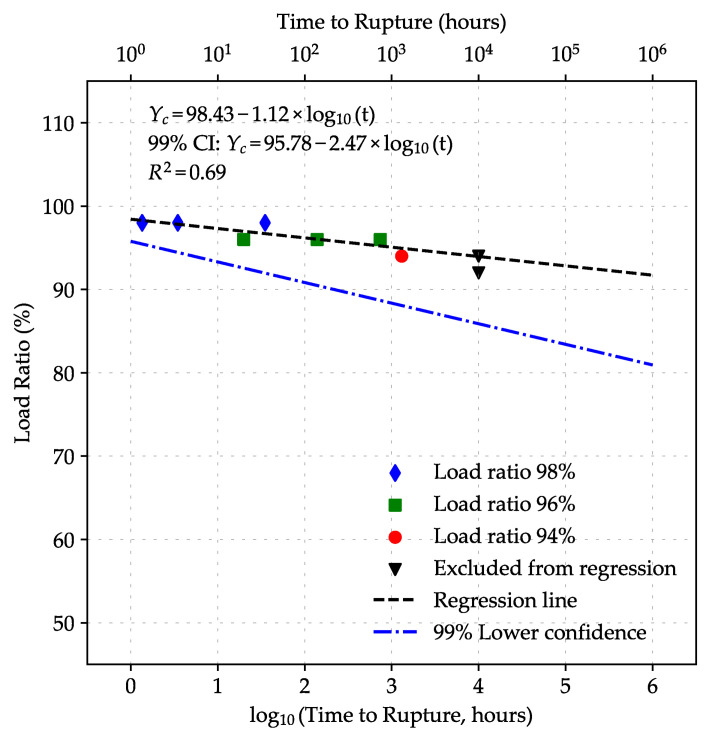
Load Ratio vs. Time to rupture for CFRP grids.

The test results summarized in Table 4 were used to create a semi-logarithmic graph, as shown in Figure 6, following the procedure detailed in Section 1.2. In this graph, the vertical axis represents the load ratio on an arithmetic scale, while the horizontal axis shows the creep rupture time on a logarithmic scale. Specimens that remained unbroken throughout the testing period are indicated by symbols (▼) in Figure 6 and were excluded from the calculation of the creep rupture regression line.

Best-fit linear curves were derived using the least squares method. The mathematical procedures for mean value regression and the 99% lower confidence interval are detailed in Appendices Appendix A and Appendix B, respectively. These analyses yield Equations (8) and (9), which describe the relationships for the mean load ratio and the 99% lower confidence interval regression of the investigated CFRP grids, respectively, as follows:

(8) $$Y_{c}\left(\%\right)=98.43-1.12\times \log_{10}\left(t\right)$$

(9) $$Y_{c,99\%CI}\left(\%\right)=95.78-2.47\times \log_{10}\left(t\right)$$

These equations were derived with a coefficient of determination ($R^{2}$) of 0.69, which is relatively high for modeling such complex phenomena.

By extrapolating the linear relationship between load ratio and creep rupture time (on a logarithmic scale) based on Equation (8), the mean extrapolated creep rupture factor for the investigated CFRP grids was projected. For service lives of 50 years and 114 years (equivalent to one million hours), the mean creep rupture factors were estimated at 92.12% and 91.71% of their ultimate static rupture load, respectively.

Similarly, the 99% lower confidence interval for the extrapolated linear relationship provided guaranteed values of the creep rupture factor, as defined by Equation (9). This interval, equivalent to minus three standard deviations (−3σ), represents the guaranteed strength of the FRP material in accordance with ACI 440.1R criteria [1]. For service lives of 50 and 114 years, the guaranteed creep rupture factors were calculated as 81.86% and 80.96% of the ultimate static rupture load, respectively.

### 3.5. Comparative Analysis

To gain a deeper understanding and validate these experimental results, a comparison can be made between the extrapolated creep rupture values for the investigated CFRP grids and those from earlier generations of CFRP composite materials. These include creep rupture studies on CFRP bars by Yamaguchi et al. [28], twist CFRP bars by Ando et al. [29], CFRP twist strands by Tokyo Rope [30], CFRP tendons by Dolan et al. [31], and CFRP strands by Grace et al. [24]. Although CFRP composite materials may differ in fiber content, resin types, and manufacturing processes, comparing their long-term creep rupture strengths can provide valuable insights. The objective of this comparison is to assess whether the creep rupture performance of earlier generations of CFRP materials, previously used in construction, is comparable to that of the CFRP grids currently under investigation.

Table 6 presents a summary of the mean experimental creep rupture strengths reported by various authors [24,28,29,30,31], all of whom used the same testing procedure and extrapolation method as employed in this study, under controlled laboratory conditions. The values obtained in this study are also included for comparison. The reported extrapolated creep rupture strength varies depending on the type of CFRP composite. For instance, smooth [26] and twisted [29] CFRP bars show significant differences in creep rupture strength, with values of 0.92 and 0.79 of their ultimate tensile strengths, respectively. This disparity can be attributed to the production process, as the twisting of bars may weaken the material due to potential fiber damage during the twisting, which reduces their creep strength. A similar trend is observed in CFRP twist strands [30] and CFRP strands [23]. Moreover, CFRP strands [23] and twisted strands [30] exhibit higher creep strength than CFRP tendons [31], as tendons, being formed from multiple twisted strands, experience additional degradation in their creep performance.

In this study, the extrapolated creep rupture strengths for the CFRP grids are 0.92 and 0.91 for service lives of 50 and 114 years, respectively. These values are attributed to the pultrusion manufacturing method used for the CFRP grids, which minimizes fiber damage compared to twisted production methods. Moreover, the extrapolation process included only specimens that reached rupture, ensuring a more precise determination of creep rupture strength. This methodology contrasts with the approach taken in a previous study [24], where unbroken specimens were included due to limited data, resulting in a lower extrapolated creep rupture factor.

Additionally, the CFRP grids in this study have a carbon fiber volume fraction of 65.4%, as detailed in Table 3. This fraction is close to the upper limit of typical CFRP composite materials reported by ACI 440.1R [1], as presented in Table 1. A high carbon fiber content likely enhances creep strength, as carbon fiber is recognized for its superior resistance to creep deformation [23,32].

### 3.6. Proposed Creep Rupture Reduction Factor

To address the effects of sustained loads and mitigate the risk of creep rupture, the creep rupture factors derived from the test program (outlined in Section 1.2) are adjusted by applying a safety margin. According to ACI 440.1R [1], which follows the Allowable Stress Design (ASD) principle, this adjustment involves applying a constant safety factor of 1.67. The experimental creep rupture strength is divided by this factor to determine the allowable stress limit under sustained loads. This allowable stress is then compared to the stress in the CFRP reinforcement, calculated using the unfactored sustained load, as outlined in Equation (5) in Section 1.1.

On the other hand, some European design standards [8,11,12] adopt a different approach by using factored loads to calculate moments and corresponding stresses in CFRP reinforcement. This method ensures that stresses remain within creep rupture limits by incorporating load uncertainties through load factors. Due to that, selecting safety factors that align with regional design philosophies is essential to guarantee safe and reliable structural performance under sustained loading conditions.

In this study, extrapolation based on the 99% lower confidence interval demonstrates that the 99% guaranteed creep rupture strength of CFRP grids over one million hours (equivalent to 114 years) is 80.96% of their ultimate tensile strength, as determined using Equation (9) in Section 3.4. Following the Allowable Stress Design (ASD) principle outlined in ACI 440.1R [1], a safety factor of 1.67 is applied to this guaranteed creep rupture strength, introducing a creep rupture reduction factor ($C_{C}$) for sustained stress design. Based on the findings of this study, a reduction coefficient of $C_{C}=0.48$ ($C_{C}=0.8096/1.67$) is proposed for the creep rupture stress limit of CFRP grids. This coefficient ensures reliability for a service life of up to 100 years while remaining consistent with ACI 440.1R’s design philosophy.

## 4. Conclusions

A comprehensive tensile creep rupture experimental program was conducted on CFRP grids under laboratory conditions. The CFRP grid specimens were continuously subjected to constant stress levels ranging from 92% to 98% of their ultimate tensile strengths for over 10,000 h (416 days). The experimental results reveal the creep characteristics of CFRP grids when exposed to high sustained stress approaching their ultimate strength. Additionally, these results allow for the extrapolation of creep rupture factors, which were compared with earlier generations of CFRP reinforcing materials. Key findings of the investigation include the following:

- The investigated CFRP grids exhibit comparable, or even slightly superior, long-term creep rupture strength when compared to previous generations of CFRP reinforcing materials.
- CFRP grids demonstrate excellent creep resistance, with specimens subjected to a sustained load of 92% remaining intact for over 10,000 h. The creep strain developed was minimal, approximately 1% to 4% of the initial strain.
- The mean creep rupture coefficients of the CFRP grids, extrapolated over 50 and 114 years (one million hours) of service life, were found to be 92.1% and 91.7% of their ultimate static tensile strength, respectively.
- The relationship between load ratio and time to rupture on a logarithmic scale has been established based on the experimental results for the investigated CFRP grid, including both the mean curve and the 99% lower confidence best-fit curve.
- Based on the obtained results, a reduction factor of 0.48 is recommended for setting the design threshold of the creep rupture stress limit for a 100-year service life, in alignment with the design philosophy of ACI 440.1R.

In conclusion, this study clarifies practical design thresholds for the tensile strength of CFRP grids in resisting sustained loading. The experimental data from creep rupture tests enhance the reference database for future studies in similar areas. Furthermore, the results demonstrate the usability of the investigated CFRP grids, particularly in their ability to resist long-term creep effects, showing performance comparable to traditional CFRP reinforcing materials.

## Figures and Tables

**Table 1:** Typical Fundamental Mechanical Tensile Properties of Steel and FRP Reinforcing Bars *.

Description	Steel Rebars	GFRP Composite	BFRP Composite	AFRP Composite	CFRP Composite
Yield Strength, MPa	276–517	N/A	N/A	N/A	N/A
Tensile Strength, MPa	483–690	483–1600	930–1380	1720–2540	600–3690
Elastic Modulus, GPa	200.0	35.0–51.0	41.0–48.5	41.0–125.0	120.0–580.0
Yield Strain (%)	0.14–0.25	N/A	N/A	N/A	N/A
Rupture Strain (%)	6.0–12.0	1.2–3.1	2.5–3.0	1.9–4.4	0.5–1.7

* The data presented in this table are sourced from ACI 440.1R [1]. The reported FRP bars have typical fiber volume fractions ranging from 0.5 to 0.7.

**Table 2:** Existing Test Methods for Tensile Creep Rupture of FRP Materials.

Reference	Year	Test Method
JSCE	1995	JSCE-E 533: Test Method for creep of continuous fiber reinforcing materials [7].
CSA	2002	CSA-S806, Annex J: Test method for creep of FRP rods [8].
ACI	2004	ACI 440.3R, B.8: Test method for creep rupture of FRP bars [9].
ASTM	2019	D7337 Standard test method for tensile creep rupture of fiber-reinforced polymer matrix composite bars [10].

Note: ASTM = American Society for Testing and Materials; ACI = American Concrete Institute; CSA = Canadian Standards Association; JSCE = Japan Society of Civil Engineers.

**Table 3:** Basic Properties of CFRP Grids Utilized in This Experiment.

Description	Detail	Remarks
Fiber Type	Carbon fiber: T700S	65.54% fiber volume
Matrix	Epoxy resin	
Glass Transition Temperature	$T_{g}$ = 252 °F/122 °C	
Grid Component Shape	Flat	
Nominal Cross-section	21.46 mm^2^	These values are taken from an average of five tested specimens.
Ultimate Load	61.14 kN	
Tensile Strength	2550.0 MPa	
Tensile Modulus	144.16 GPa	
Ultimate Strain	19,770.30 με (1.98%)	

**Table 4:** Summary of Tensile Creep Rupture Test Results for CFRP Grids.

Load Ratio (%)	Target Load (kN)	Specimen Marks	Initial Strain (με)	Rupture Time (h)	Rupture Strain (με)	Creep Strain ^+^ (με)
98.0%	59.92	C-98-01	18,978.0	3.47	20,385.0	1407.0
		C-98-02	18,875.0	1.35	20,209.0	1334.0
		C-98-03	18,911.0	35.03	19,790.0	879.0
96.0%	58.69	C-96-01	18,449.0	138.97	20,702.0	2206.0
		C-96-02	18,471.0	739.87	21,053.0	2582.0
		C-96-03	18,391.0	19.17	19,323.0	932.0
94.0%	57.47	C-94-01 *	17,962.0	–	–	–
		C-94-02	17,853.0	1303.27	20,188.0	2335.0
		C-94-03 *	17,810.0	–	–	–
92.0%	56.25	C-92-01 *	17,584.0	–	–	–
		C-92-02 *	17,739.0	–	–	–
		C-92-03 *	17,618.0	–	–	–

^+^ The creep strains were calculated by subtracting the rupture strain from the initial strain. * Unbroken specimens were terminated after 10,000 h, and performed a tensile test for evaluating the retention properties.

**Table 5:** Mechanical Properties of CFRP Grids After Creep Rupture Testing.

Specimen	Creep Strain ^+^ (με)	Tensile Strength (MPa)		Ultimate Strain (με)		Elastic Modulus (GPa)	
		**Value**	**Retention (%)**	**Value**	**Retention (%)**	**Value**	**Retention (%)**
Reference *		2849.40	–	19,770.30	–	144.16	–
C-94-01	613.0	2902.60	101.9%	18,755.55	94.9%	154.76	107.4%
C-94-03	262.0	2803.77	98.4%	19,578.53	99.0%	143.21	99.3%
C-92-01 **	537.0	2877.66	100.9%	–	–	–	–
C-92-02 **	841.0	2950.66	103.6%	–	–	–	–
C-92-03	723.0	2816.37	98.8%	20,391.33	103.1%	138.12	95.8%
**Average**	–	2870.21	100.7%	19,575.14	99.0%	145.36	100.8%

^+^ Creep strains were determined by subtracting the initial strain from the final strain. * The reference value represents the average strain of the original specimens prior to the creep test. ** Strain gauge was damaged during specimen removal; strain data are unavailable for this specimen.

**Table 6:** Mean Extrapolated Creep Rupture Factor of CFRP Materials Reported by Various Authors.

Reference	Year	Remarks	Extrapolated Creep Rupture Strength		Test Endurance
			**At 50 Years**	**At 114 Years**	
Yamaguchi et al. [28]	1997	CFRP bar	0.92	–	100 h
Ando et al. [29]	1998	Twist CFRP bar	0.79	–	100 h
Tokyo Rope [30]	2000	CFRP twist strand	–	0.85	–
Dolan [31]	2001	CFRP tendons	–	0.70	12,000 h
Grace et al. [24]	2023	CFRP strands	–	0.88	1000 h
This study	2024	CFRP grids	0.92	0.91	10,000 h

## Data Availability

The data presented in this study are available on request from the corresponding author. The data are not publicly available due to privacy reasons.

## Appendix A. Derivation of the Best-Fit Linear Curve Using Least Squares

The best-fit linear curve, plotted on a semi-logarithmic scale, representing the relationship between the load ratio and the time to rupture of FRP materials is expressed as follows:

(A1) $$Y_{c}=a_{1}-b_{1}\times \log_{10}\left(t\right),$$

where $Y_{c}$ represents the load ratios and *t* denotes the corresponding rupture times, both derived from the long-term creep rupture tests in Table 4. The parameters $a_{1}$ and $b_{1}$ are to be determined.

1. Transform the Model:
  Let $x=\log_{10}\left(t\right)$ and $y=Y_{c}$. The equation then becomes
  (A2) $$y=a_{1}+b_{2}x,\;\mathrm{where}\;b_{2}=-b_{1},$$
2. Least Squares Objective:
  To find the best-fit values for $a_{1}$ and $b_{2}$, we minimize the sum of squared errors (the loss function):
  (A3) $$L=\sum_{i=1}^{n}{\left(y_{i}-\left(a_{1}+b_{2}x_{i}\right)\right)}^{2}.$$
3. Compute the Normal Equations:
  Expand the expression for L:
  (A4) $$L=\sum_{i=1}^{n}{\left(y_{i}-a_{1}-b_{2}x_{i}\right)}^{2}.$$
  Next, take partial derivatives of L with respect to $a_{1}$ and $b_{2}$, and set them to zero to minimize the error.
  - With respect to $a_{1}$:
  (A5) $$\frac{\partial L}{\partial a_{1}}=-2\sum_{i=1}^{n}\left(y_{i}-a_{1}-b_{2}x_{i}\right)=0,$$
  Simplifying gives the following:
  (A6) $$\sum_{i=1}^{n}y_{i}=na_{1}+b_{2}\sum_{i=1}^{n}x_{i}.$$
  - With respect to $b_{2}$:
  (A7) $$\frac{\partial L}{\partial b_{2}}=-2\sum_{i=1}^{n}x_{i}\left(y_{i}-a_{1}-b_{2}x_{i}\right)=0,$$
  Simplifying gives the following:
  (A8) $$\sum_{i=1}^{n}x_{i}y_{i}=a_{1}\sum_{i=1}^{n}x_{i}+b_{2}\sum_{i=1}^{n}x_{i}^{2}.$$
4. Solve for Parameters:
  Solving the system of Equations (A6) and (A8) yields the parameters $a_{1}$ and $b_{2}$. Subsequently, $b_{1}$ is computed as $b_{1}=-b_{2}$. Finally, substituting the values of $a_{1}$ and $b_{1}$ into Equation (A1) gives the best-fit linear curve.

## Appendix B. Derivation of Best-Fit Curve with the 99% Lower Confidence Interval

This appendix outlines the procedure to compute the 99% lower confidence interval for the parameters $a_{1}$ and $b_{2}$ in the regression model.

1. Model Overview:
  The regression model is expressed as follows:
  (A9) $$y_{i}=a_{1}+b_{2}x_{i}+\epsilon_{i},\;\epsilon_{i}∼N\left(0,\sigma^{2}\right),$$
  where
  - $y_{i}=Y_{c}$: Observed load ratio.
  - $x_{i}=\log_{10}\left(t_{i}\right)$: Log-transformed rupture time.
  - $a_{1},b_{2}$: Regression coefficients ($b_{2}=-b_{1}$).
  - $\epsilon_{i}$: Independent random errors.
2. Estimate Variance of Coefficients:
  Using the least squares method, the variance–covariance matrix for the estimated parameters $\left[a_{1},b_{2}\right]$ is given by the following:
  (A10) $$\mathrm{Var}\left(\hat{\beta }\right)=\sigma^{2}{\left(X^{⊤}X\right)}^{-1},$$
  where
  - X: The design matrix, which includes a column of ones for $a_{1}$ and a column of $x_{i}$ values for $b_{2}$.
  - $\sigma^{2}$: The estimated variance of residuals, computed as follows:
    (A11) $$\sigma^{2}=\frac{1}{n-2}\sum_{i=1}^{n}{\left(y_{i}-{\hat{y}}_{i}\right)}^{2},$$
  The diagonal elements of $\mathrm{Var}(\hat{\beta })$ provide the variances of ${\hat{a}}_{1}$ and ${\hat{b}}_{2}$.
3. Confidence Interval for Parameters:
  The confidence interval for each parameter $\beta_{k}$ (where *k* is either $a_{1}$ or $b_{2}$) is computed as follows:
  (A12) $${\hat{\beta }}_{k}\pm t_{\alpha /2,n-2}\cdot \sqrt{\mathrm{Var}({\hat{\beta }}_{k})},$$
  where
  - $t_{\alpha /2,n-2}$: The critical value from the *t*-distribution for n−2 degrees of freedom at α=0.01.
  - $\sqrt{\mathrm{Var}({\hat{\beta }}_{k})}$: The standard error of the parameter estimate.
  For the 99% lower confidence limit, the interval is truncated at the lower bound:
  (A13) $$\beta_{k}^{\mathrm{lower}}={\hat{\beta }}_{k}-t_{0.005,n-2}\cdot \sqrt{\mathrm{Var}({\hat{\beta }}_{k})}.$$
4. Transform Back to Original Parameters:
  Since $b_{1}=-b_{2}$, the 99% lower confidence interval for $b_{1}$ is obtained by applying the transformation to the bounds of $b_{2}$:
  (A14) $$b_{1}^{\mathrm{lower}}=-b_{2}^{\mathrm{upper}}.$$
5. Final Best-Fit Equation with Confidence Intervals:
  The final best-fit linear equation, including the 99% lower confidence intervals for $a_{1}$ and $b_{1}$, is expressed as follows:
  (A15) $$Y_{c}=a_{1}^{\mathrm{lower}}-b_{1}^{\mathrm{lower}}\cdot \log_{10}\left(t\right).$$

## References

[1] (2015). Guide for the Design and Construction of Structural Concrete Reinforced with Fiber-Reinforced Polymer (FRP) Bars.

[2] Belarbi A., Dawood M., Mirmiran A., Bowman M. (2017). Synthesis of Concrete Bridge Piles Prestressed with CFRP Systems.

[3] (2007). Report on Fiber-Reinforced Polymer (FRP) Reinforcement for Concrete Structures.

[4] Vijayan D.S., Sivasuriyan A., Devarajan P., Stefańska A., Wodzyński Ł., Koda E. (2023). Carbon Fibre-Reinforced Polymer (CFRP) Composites in Civil Engineering Application—A Comprehensive Review. Buildings.

[5] Pawlak A.M., Górny T., Dopierała Ł., Paczos P. (2022). The Use of CFRP for Structural Reinforcement—Literature Review. Metals.

[6] ISIS Canada (2006). Specifications for Product Certification on Fibre Reinforced Polymers (FRPs) as Internal Reinforcement in Concrete Structures.

[7] (1995). Test Method for Creep Failure of Continuous Fiber Reinforcing.

[8] (2012). Design and Construction of Building Components with Fibre-Reinforced Polymers.

[9] (2012). Guide Test Methods for Fiber-Reinforced Polymer Composites for Reinforcing or Strengthening Concrete and Masonry Structures.

[10] (2019). Standard Test Method for Tensile Creep Rupture of Fiber Reinforced Polymer Matrix Composite Bars.

[11] (2006). Guide for Design and Construction of Concrete Structures Reinforced with Fiber-Reinforced Polymers Bars.

[12] (2007). FRP Reinforcement in RC Structures.

[13] (1995). Recommendation for Design and Construction of Concrete Structures Using Continuous Fiber Reinforcing Materials.

[14] AASHTO (2018). Guide Specifications for the Design of Concrete Bridge Beams Prestressed with Carbon Fiber-Reinforced Polymer (CFRP) Systems.

[15] Patnaik S.S., Roy T. (2021). Viscoelastic and mechanical properties of CNT-reinforced polymer-based hybrid composite materials using hygrothermal creep. Polym. Polym. Compos..

[16] Ornaghi H.L., Almeida J.H.S., Monticeli F.M., Neves R.M. (2020). Stress relaxation, creep, and recovery of carbon fiber non-crimp fabric composites. Compos. Part C Open Access.

[17] Ornaghi H.L., Almeida J.H.S., Monticeli F.M., Neves R.M., Cioffi M.O.H. (2021). Time-temperature behavior of carbon/epoxy laminates under creep loading. Mech. Time-Depend. Mater..

[18] Santos P., Silva A.P., Reis P. (2023). Effect of carbon nanofibers on the viscoelastic response of carbon/epoxy composites. J. Reinf. Plast. Compos..

[19] Almeida J.H.S., Ornaghi H.L., Lorandi N.P., Bregolin B.P., Amico S.C. (2018). Creep and interfacial behavior of carbon fiber reinforced epoxy filament wound laminates. Polym. Compos..

[20] Majda P., Skrodzewicz J. (2009). A modified creep model of epoxy adhesive at ambient temperature. Int. J. Adhes. Adhes..

[21] Okuka A.S., Zorica D. (2020). Fractional Burgers models in creep and stress relaxation tests. Appl. Math. Model..

[22] Ascione F., Berardi V.P., Feo L., Giordano A. (2008). An experimental study on the long-term behavior of CFRP pultruded laminates suitable to concrete structures rehabilitation. Compos. Part B Eng..

[23] Yang D., Zhang J., Song S., Zhou F., Wang C. (2018). Experimental Investigation on the Creep Property of Carbon Fiber Reinforced Polymer Tendons under High Stress Levels. Materials.

[24] Grace N.F., Mohamed M.E., Bebawy M.R. (2023). Evaluating fatigue, relaxation, and creep rupture of carbon-fiber-reinforced polymer strands for highway bridge construction. PCI J..

[25] (2017). Standard Test Method for Tensile Properties of Polymer Matrix Composite Materials.

[26] Rossini M., Saqan E., Nanni A. (2019). Prediction of the creep rupture strength of GFRP bars. Constr. Build. Mater..

[27] Wang X., Shi J., Liu J., Yang L., Wu Z. (2014). Creep behavior of basalt fiber reinforced polymer tendons for prestressing application. Mater. Des..

[28] Yamaguchi T., Kato Y., Nishimura T., Uomoto T. (1997). Creep Rupture of FRP Rods Made of Aramid, Carbon, and Glass Fibers. Proceedings of the Third International Symposium on Non-Metallic (FRP) Reinforcement for Concrete Structures (FRPRCS-3).

[29] Ando N., Matsukawa H., Fujii M., Miyagawa T., Inoue S. (1998). Experimental Studies on the Long-Term Tensile Properties of FRP Tendons. Fracture Mechanics of Concrete Structures, Proceedings of the FRAMCOS-3, Gifu, Japan, 12–16 October 1998.

[30] Tokyo Rope (2000). Carbon Fiber Composite Cable.

[31] Dolan C.W., Hamilton H.R., Bakis C.E., Nanni A. (2001). Design Recommendations for Concrete Structures Prestressed with FRP Tendons.

[32] (2004). Prestressing Concrete Structures with FRP Tendons.

